# Complex Mutations & Subpopulations of Deletions at Exon 19 of EGFR in NSCLC Revealed by Next Generation Sequencing: Potential Clinical Implications

**DOI:** 10.1371/journal.pone.0042164

**Published:** 2012-07-27

**Authors:** Antonio Marchetti, Maela Del Grammastro, Giampaolo Filice, Lara Felicioni, Giulio Rossi, Paolo Graziano, Giuliana Sartori, Alvaro Leone, Sara Malatesta, Michele Iacono, Luigi Guetti, Patrizia Viola, Felice Mucilli, Franco Cuccurullo, Fiamma Buttitta

**Affiliations:** 1 Center of Predictive Molecular Medicine, Center of Excellence on Aging, University-Foundation, Chieti, Italy; 2 Anatomic Pathology Unit, Azienda Ospedaliero-Universitaria Policlinico of Modena, Modena, Italy; 3 Anatomic Pathology Unit, San Camillo-Forlanini Hospital, Rome, Italy; 4 Applied Science - Roche Diagnostics, Monza, Italy; 5 Department of Surgery, University of Chieti, Chieti, Italy; 6 Oncological and Cardiovascular Molecular Medicine Unit, University-Foundation, Chieti, Italy; National Taiwan University Hospital, Taiwan

## Abstract

Microdeletions at exon 19 are the most frequent genetic alterations affecting the Epidermal Growth Factor Receptor (EGFR) gene in non-small cell lung cancer (NSCLC) and they are strongly associated with response to treatment with tyrosine kinase inhibitors. A series of 116 NSCLC DNA samples investigated by Sanger Sequencing (SS), including 106 samples carrying exon 19 EGFR deletions and 10 without deletions (control samples), were subjected to deep next generation sequencing (NGS). All samples with deletions at SS showed deletions with NGS. No deletions were seen in control cases. In 93 (88%) cases, deletions detected by NGS were exactly corresponding to those identified by SS. In 13 cases (12%) NGS resolved deletions not accurately characterized by SS. In 21 (20%) cases the NGS showed presence of complex (double/multiple) frameshift deletions producing a net in-frame change. In 5 of these cases the SS could not define the exact sequence of mutant alleles, in the other 16 cases the results obtained by SS were conventionally considered as deletions plus insertions. Different interpretative hypotheses for complex mutations are discussed. In 46 (43%) tumors deep NGS showed, for the first time to our knowledge, subpopulations of DNA molecules carrying EGFR deletions different from the main one. Each of these subpopulations accounted for 0.1% to 17% of the genomic DNA in the different tumors investigated. Our findings suggest that a region in exon 19 is highly unstable in a large proportion of patients carrying *EGFR* deletions. As a corollary to this study, NGS data were compared with those obtained by immunohistochemistry using the 6B6 anti-mutant EGFR antibody. The immunoreaction was E746-A750del specific. In conclusion, NGS analysis of EGFR exon 19 in NSCLCs allowed us to formulate a new interpretative hypothesis for complex mutations and revealed the presence of subpopulations of deletions with potential pathogenetic and clinical impact.

## Introduction

Lung cancer is the leading cause of cancer-related deaths in western countries and standard therapeutic strategies including surgery, chemotherapy, and radiotherapy have almost reached a plateau [1]. In recent years, the pharmacological treatment of non-small cell lung cancer (NSCLC) has undergone a major contribution by the introduction of new molecular targeted drugs whose effectiveness is closely dependent on the presence of specific genetic mutations in the tumor context [2]–[6]. Somatic mutations in the tyrosine kinase domain of the Epidermal Growth Factor Receptor (*EGFR*) gene emerged as one of the most relevant targets for lung cancer treatment [7]–[12]. Most of *EGFR* mutations are in-frame microdeletions at exon 19 affecting the conserved amino acids ELREA. These mutations represent 44% to 80% of *EGFR* mutations in different studies [13] and they are strongly associated with sensitivity to tyrosine kinase inhibitors [14]–[16]. Exon 19 deletions usually affect one allele, with the other one being wild type. The technique most widely used to detect and characterize *EGFR* deletions is Sanger sequencing (SS) of an exon 19 PCR product [17], [18]. The presence of wild type DNA amplified from the normal exon 19 allele may hamper an accurate detection of the microdeletion in the mutant allele even if the best sequence alignment algorithms are used. DNA-cloning in plasmids followed by sequencing of multiple clones can allow a more accurate analysis of deletions, especially in case of complex mutations. Since DNA-cloning and sequencing is time consuming this approach has been rarely used [19], [20].

Massive parallel sequencing, also known as next generation sequencing (NGS), could be particularly suited for the detection of microdeletions. This new technology, based on PCR from single molecules before sequencing, realizes a sort of chemical cloning. Therefore, wild type and mutant alleles are analyzed separately, resorting in an accurate characterization of mutations. The high accuracy of NGS technologies is also achieved by multiple read coverage of a variant base in an individual sample [21]–[24].

These particular features make the NGS one of the most sensitive technology currently available for mutation scanning, allowing to detect somatic mutations in subpopulations of DNA molecules, as shown in dilution experiments [25], [26].

We decide to investigate a large number of somatic microdeletions of the *EGFR* gene by deep sequencing. Results were compared with those obtained by SS and potential biological and clinical implications are highlighted.

## Results

A series of 116 NSCLC DNA samples investigated by SS, including 106 samples carrying exon 19 *EGFR* deletions and 10 samples without deletions (control samples), were subjected to deep NGS. About 440.000 sequences, with a mean of 3497+/−158 sequences per samples, for a total of about 72.000.000 bp, were obtained.

**Figure 1:**
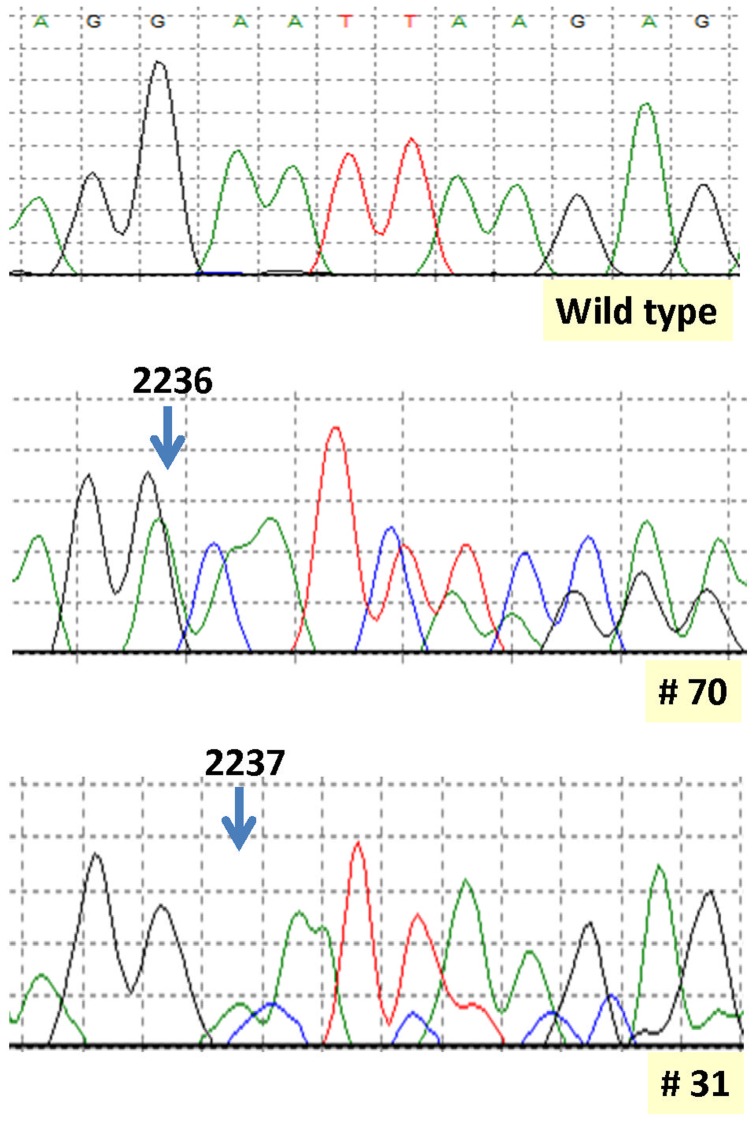
Sanger sequencing (SS) analysis of two mutated cases (#70 and #31) compared with a wild type reference DNA. Wild type and deleted alleles are superimposed in SS electropherograms. In case #70, carrying a 2236–2250del, the peaks are perfectly aligned and the starting point of the deletion at base 2236 is easily detectable. Next generation sequencing (NGS) confirmed this type of deletion. In case #31, carrying the same mutation, as detected by NGS, peaks in the SS electropherogram are not well aligned and the starting point of the deletion was incorrectly positioned by the operator at base 2237.

All the samples with deletions at SS were found to be positive for exon 19 deletions with NGS. No deletions were observed in control cases. Deletions detected by SS were exactly confirmed in 93 (88%) cases and the frequency of concordant data was not statistically different in the three centers. In 13 cases (12%) the deletion was not correspondent to that observed by SS where, in 6 cases the starting-ending point was not correctly detected and the deletion was misinterpreted (i.e. c2235–2249del instead of c2236–2250del and viceversa or 2237_2251del instead of 2236_2250del, as reported in Figure 1), in 7 cases the starting-ending point of the deletions were clear, but the exact sequence of the deleted bases could not be accurately defined (Table 1).

**Table 1:** Cases in which the accurate sequence of deleted bases was incorrectly determined or not assessable by Sanger sequencing compared with data obtained by Next generation sequencing.

Case	Sanger Sequencing	Next Generation Sequencing	AA change
	Mutation	Mutation	
# 1	NA	c.2240_2254del	p.L747_T751del
# 10	NA	c.2238_2249del c.2252_2253CA>AT	p.L746_A750del; T751N
# 31	c.2237_2251del	c.2236_2250del	p.E746_A750del
# 35	c.2235_2249del	c.2236_2250del	p.E746_A750del
# 45	c.2236_2250del	c.2235_2249del	p.E746_A750del
# 47	NA	c.2237_2253del c.2255del	p.E746_S752>V
# 49	c.2235_2249del	c.2236_2250del	p.E746_A750del
# 51	NA	c.2237_2253del c.2255del	p.E746_S752>V
# 53	NA	c.2239_2248del c.2253_2260del	p.L747_K754>QQ^a^
# 54	c.2235_2249del	c.2236_2250del	p.E746_A750del
# 59	NA	c.2230_2237del c.2245_2251del	p.I744_A750>IK^a^
# 60	c.2235_2249del	c.2236_2250del	p.E746_A750del
# 61	NA	c.2239_2262delc.2263_2274dup(ins)	p.L747_K754>ANKE^a^

Abbreviations: NA, the exact sequence of deleted bases could not be accurately defined (not assessable).

a Novel mutation.

**Figure 2:**
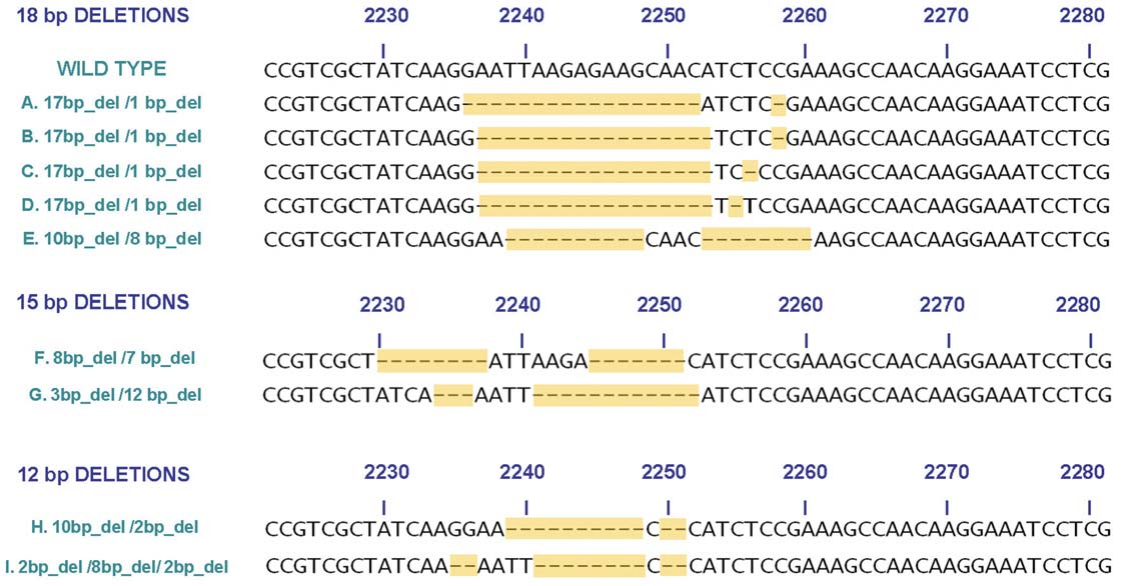
Double/multiple deletions in exon 19 of EGFR revealed by the 454 GS Junior system. Complex mutations are grouped according to the length of the deletion. Deleted bases are indicated by dashes highlighted in grey. Correspondence with numbers of tumor samples: A (#22); B (#14, #67); C (#25); D (#12, #16, #24, #38, #39, #44, #47, #51, #73, #74, #81, #89); E (#53); F (#59); G (#64); H (#38); I (#95).

**Figure 3:**
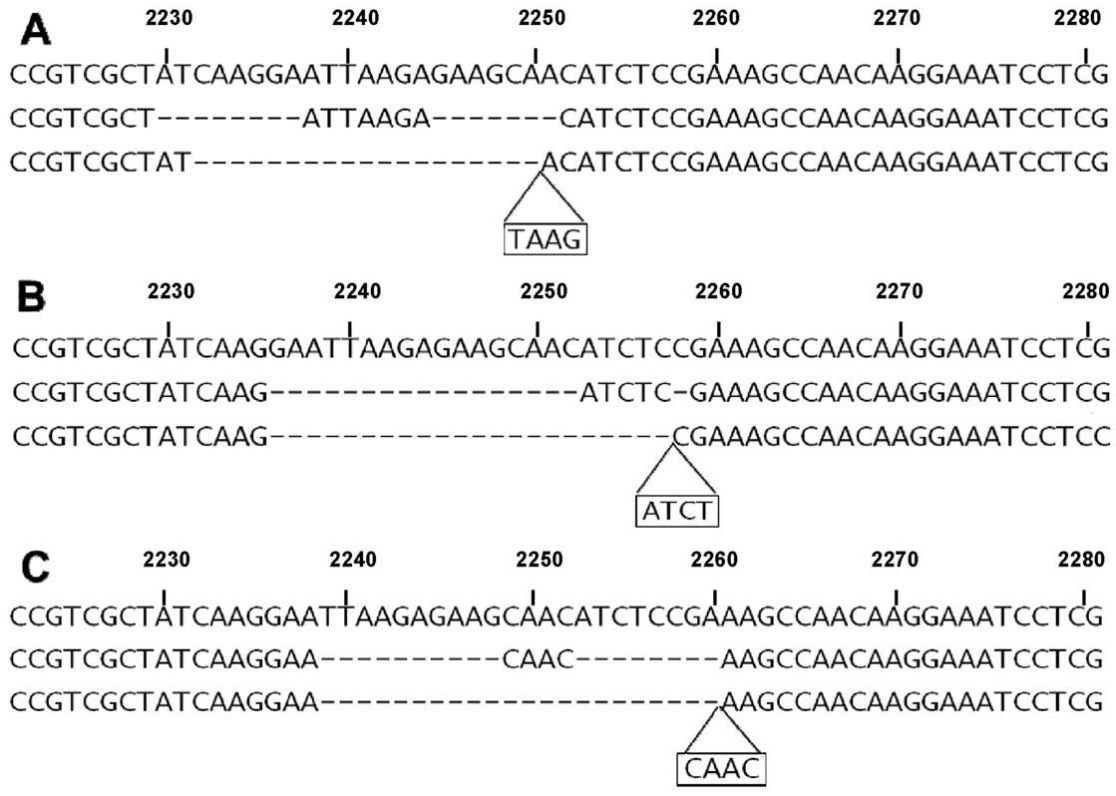
Different interpretation of sequence data in case of complex mutations. A complex in frame deletion of 15 bp (A) can be considered as composed of two non-in frame deletions of 8 bp and 7 bp separated by a consensus sequence of 7 bp, in other words a double deletion. Conventionally, this mutation could have been interpreted as the sum of a non-in frame deletion of 19 bp and non-in frame insertion of 4 bp. Accordingly, in B and C are reported different interpretations for mutations giving rise to complex in frame deletions of 18 bps. Deleted bases are indicated by dashes. Inserted bases are reported a in a frame under the sequence. Correspondence with numbers of tumor samples: A (#59); B (#22); C (#53).

In 21 cases (20%) the AVA software of the 454 Junior instrument showed the presence of double or multiple frameshift deletions that produced a net in-frame change. Most of these complex mutations (16 cases) were the sum of a long and short non-in frame deletion (17 bp_del+1 bp_del). A double deletion of 18 bp (10 bp_del+8 bp_del), two double deletions of 15 bp (12 bp_del+3 bp_del and 8 bp_del+7 bp_del), and a triple deletion of 12 bp (2 bp_del+8 bp_del+2b p_del) were also observed (Figure 2). In addition to these multiple deletions, a case showing a novel, particularly complex mutation, composed of a 24 bp_del followed by a duplication-insertion of 12 bp was also seen. In 5 (24%) of these cases carrying complex mutations the SS did not allow to define the exact sequence of the mutant alleles (Table 1), in the other 16 (14%) cases the alterations obtained by SS were interpreted by the operators as complex deletions (deletions+insertions), according to previously reported data [19], [27], [28]. A comparison between SS and NGS data is reported in Table 2. Figure 3 shows selected cases exemplifying the new interpretation of data by the 454 Life Sciences software compared with conventional interpretation.

**Table 2:** Comparison of data obtained by Sanger Sequencing and Next Generation Sequencing analysis on cases carrying complex deletions.

Case	Sanger Sequencing		Next Generation Sequencing		AA change
	Mutation	Rearrangement	Mutation	Rearrangement	
# 22	c.2236_2257delinsATCT	22 bp_del/4 bp_ins	c.2236_2252del c.2258del	17 bp_del/1 bp_del	p.E746_P753>IS^a^
# 14	c.2237_2257delinsTCT	21 bp_del/3 bp_ins	c.2237_2253del c.2258del	17 bp_del/1 bp_del	p.E746_P753>VS
# 67	c.2237_2257delinsTCT	21 bp_del/3 bp_ins	c.2237_2253del c.2258del	17 bp_del/1 bp_del	p.E746_P753>VS
# 25	c.2237_2256delinsTC	20 bp_del/2 bp_ins	c.2237_2253del c.2256del	17 bp_del/1 bp_del	p.E746_S752>V
# 12	c.2237_2255delinsT	19 bp_del/1 bp_ins	c.2237_2253del c.2255del	17 bp_del/1 bp_del	p.E746_S752>V
# 16	c.2237_2255delinsT	19 bp_del/1 bp_ins	c.2237_2253del c.2255del	17 bp_del/1 bp_del	p.E746_S752>V
# 24	c.2237_2255delinsT	19 bp_del/1 bp_ins	c.2237_2253del c.2255del	17 bp_del/1 bp_del	p.E746_S752>V
# 38	c.2237_2255delinsT	19 bp_del/1 bp_ins	c.2237_2253del c.2255del	17 bp_del/1 bp_del	p.E746_S752>V
# 39	c.2237_2255delinsT	19 bp_del/1 bp_ins	c.2237_2253del c.2255del	17 bp_del/1 bp_del	p.E746_S752>V
# 44	c.2237_2255delinsT	19 bp_del/1 bp_ins	c.2237_2253del c.2255del	17 bp_del/1 bp_del	p.E746_S752>V
# 73	c.2237_2255delinsT	19 bp_del/1 bp_ins	c.2237_2253del c.2255del	17 bp_del/1 bp_del	p.E746_S752>V
# 74	c.2237_2255delinsT	19 bp_del/1 bp_ins	c.2237_2253del c.2255del	17 bp_del/1 bp_del	p.E746_S752>V
# 81	c.2237_2255delinsT	19 bp_del/1 bp_ins	c.2237_2253del c.2255del	17 bp_del/1 bp_del	p.E746_S752>V
# 89	c.2237_2255delinsT	19 bp_del/1 bp_ins	c.2237_2253del c.2255del	17 bp_del/1 bp_del	p.E746_S752>V
# 64	c.2235_2252delinsATT	18 bp_del/3 bp_ins	c.2234_2236del c.2241_2252del	3 bp_del/12 bp_del	p.K746_T751>L^a^
# 95	c.2235_2251delinsAATTC	17 bp_del/5 bp_ins	c.2235_2236delc.2241_2248delc.2250_2251del	2 bp_del/8 bp_del/2 bp_del	p.E746_T751>IP

a Novel mutation.

Seventeen (81%) of 21 double/multiple deletions resort in a loss of 18 bp. Among the 28 deletions of 18 bp in this series, 17 (61%) were double deletions, whereas only 2 (3%) of 74 deletions of 15 bp were double/multiple (P<0,000001).

Five of the complex mutations observed in the present study were considered novel mutations, as they were never reported before (cases indicated by an asterisk* in Table 1 and 2). In 3 (60%) of these cases the SS was unable to accurately detect the mutations.

Selected cases with different simple and complex deletions were investigated by immunohistochemistry with monoclonal antibody 6B6 anti-mutant EGFR [EGF Receptor (E746-A750del Specific) (6B6) XP® Rabbit mAb #2085 (Cell Signaling)] that recognize EGFR proteins with exon 19 deletions. A strong immunohistochemical staining (2+/3+) was seen in tumors carrying the 2235–2249del and 2236–2250del (E746-A750del). No immunohistochemical signal was present in tumors with other simple or complex deletions (Table 3).

**Table 3:** Immunohistochemical staining with deletion specific 6B6 monoclonal antibody in Non-Small Cell Lung Cancers carrying different deletions at exon 19 of the *EGFR* gene detected by Next Generation Sequencing.

Case	Deletion	Codon	Score^a^
#3	c.2235_2249del15	p.E746_A750del	1+/2+
#4	c.2235_2249del15	p.E746_A750del	3+
#5	c.2235_2249del15	p.E746_A750del	2+
#11	c.2235_2249del15	p.E746_A750del	3+
#15	c.2235_2249del15	p.E746_A750del	2+
#17	c.2235_2249del15	p.E746_A750del	3+/2+
#19	c.2235_2249del15	p.E746_A750del	3+/2+
#28	c.2235_2249del15	p.E746_A750del	2+/3+
#2	c.2236_2250del15	p.E746_A750del	1+/2+
#12	c.2237_2253del17c.2255del	p.E746_S752>V	0
#14	c.2237_2253del17c.2258del	p.E746_S752>VS	0
#16	c.2237_2253del17c.2255del	p.E746_S752>V	0
#10	c.2238_2249del12c.2252_2253CA>AT	p.L746_A750del; T751N	0
#18	c.2239_2248del10 insC	p.L747_A750del>P	0
#1	c.2240_2254del15	p.L747_T751del	0
#9	c.2240_2254del15	p.L747_T751del	0
#20	c.2240_2254del15	p.L747_T751del	0

a The score system is described in detail in Materials and Methods.

**Figure 4:**
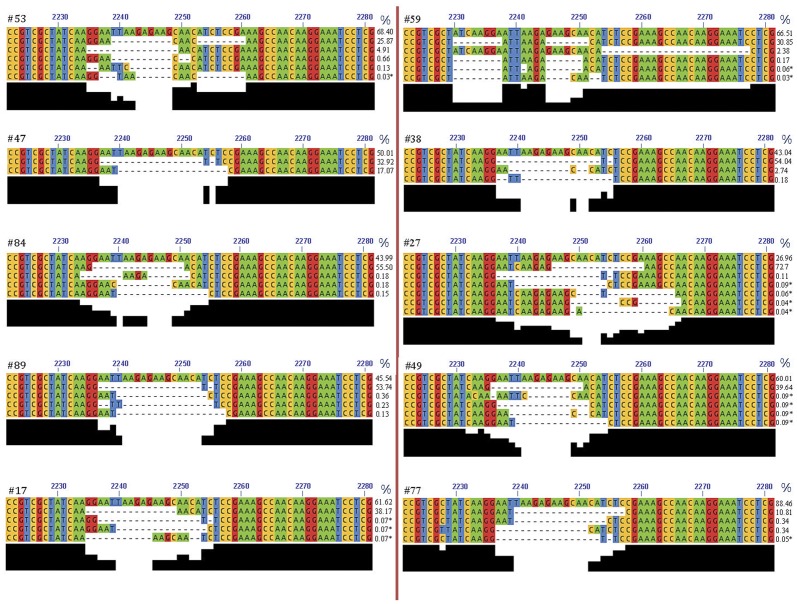
Examples of subpopulations of EGFR deletions at exon 19. The Figure reports 10 selected cases of tumors (#53, #59, #47, #38, #84, #27, #89, #49, #17, #77) showing subpopulations of EGFR deletions at exon 19. In each case the first line corresponds to the wild type sequence. The different bases are highlighted by different colours. Deleted bases are reported as dashes. The black bars under the sequences indicate the consensus for the different bases involved in deletions. On the right of each case is reported the percentage with which the wild type and deleted molecules were present in tumor DNA. The number (N) of sequences obtained in each case were as follow: #53 (N = 6.484), #59 (N = 5.835), #47 (N = 5.629), #38 (N = 4.776), #84 (N = 2.641), #27 (N = 4.389), #89 (N = 3.855), #49 (N = 2.172), #17 (N = 2.856), #77 (N = 3.526). Cases carrying subpopulations in less than 0.1% of the DNA molecules are labeled with an asterisk.

In 70 (66%) tumors the NGS analysis revealed, in addition to the main deletion, the presence of subpopulations of DNA molecules carrying different deletions which, in most cases, were structurally related to the main one. Each of these deletions accounted for 0.03% to 17% of the genomic DNA in the different tumors investigated. Most (69%) of the deletions observed in subpopulations of DNA molecules were in frame, in 31% of cases minimally expanded non-in frame deletions were seen. In the cases with subpopulations of deletions, PCR amplification and NGS analysis were repeated using the same experimental conditions. We confirmed all the subpopulations present in at least the 0.1% of the DNA molecules. Instead, we were able to confirm the data in only a portion (about 40%) of cases carrying subpopulations in less than 0.1% of the DNA molecules (data not shown). Aware of the fact that it is theoretically very difficult to confirm, in independent PCRs, the presence of subpopulations of deletions if they are present in few readouts, we decided to consider these data as low confidence events. Therefore, we divided the subpopulations in high confidence events, when they were present in at least the 0.1% and low confidence events when they occurred in less than 0.1% of the DNA. Forthy-six tumors (43%) showed high confidence subpopulations of deletions. Four tumors (4%) showed substantial subpopulations of deletions with at least one of them representing more than 2% of genomic DNA. These substantial subpopulations were present in 4 (19%) of 21 cases in which the main mutation was a double/multiple deletion and in none of the 85 cases carrying simple deletions as main mutations (P<0.001). A selection of cases carrying subpopulations of deletions is shown in Figure 4. Table S2 reports all the *EGFR* mutations observed by NGS in the whole series of tumors investigated.

## Discussion

The present study was devised to evaluate a large series of microdeletions at exon 19 of the *EGFR* gene by the 454 GS Junior system. This new technical approach is based on the amplification of single DNA molecules by emulsion PCR giving rise to a sort of chemical cloning before pyrosequence analysis. Since expanded wild type and mutant alleles are examined separately, the method can allow an accurate evaluation of mutations without the interference of wild type alleles as it occurs when conventional sequencing methods are used. The results of NGS were compared with those obtained by conventional SS. In about 12% of the samples analyzed, SS failed in finding the accurate sequence of deleted bases, either using a dedicated software or manually. In particular, in 6 cases, the starting-ending point of the deletion point was not correctly detected. This can be ascribed to the fact that, by using Sanger sequencing, the wild type and mutated alleles are superimposed on the same electropherogram. In some samples, it can happen that the alignment of the two alleles is not perfect, as shown in Figure 1, so that the operator can misinterpret the starting-ending point of the deletion. In other 7 cases, the starting-ending point of the deletions were clear but the exact sequence of the mutant alleles was partially obscured (not assessable).

An accurate detection of *EGFR* microdeletions is highly recommendable, in that different deletions could have different effect on tumor development and progression or patient outcome after treatment with EGFR TKIs. Moreover, different deletion may have specific effects on the antigenicity of proteins carrying deletion. In our study, we have tested this hypothesis by evaluating the possibility to detect *EGFR* deletions with specific monoclonal antibodies in tumors with different deletions accurately characterized by NGS. We have shown that the 6B6 anti-mutant EGFR antibody strongly reacts to EGFR proteins carrying the E746_A750del, whereas the immunoreaction was absent in tumors affected by other types of deletions. Our data are in agreement with previously published data obtained with this antibody in larger series of NSCLCs [29], [30]. However, due to the limited number of cases examined in these reports, additional studies will be required to definitely clarify this point. Our results suggest that the development of new monoclonal antibodies or cocktails of antibodies would require the exact knowledge of the deletions and that NGS could be a very accurate and reliable technique to address this point.

The alignment of the numerous sequences obtained by NGS by dedicated softwares, allowed to formulate a new interpretative hypothesis on the nature of particular *EGFR* deletions. A series of frequent (20% of cases) complex deletions, most of which reported as deletions associated to insertions in previous reports [19], [27], [28], may also be ascribed to the presence of non-in frame double or multiple deletions producing a net in-frame loss of genetic material. This interpretation is in keeping with the hypothesis that a short region within exon 19 is particularly fragile and preferentially subjected to microdeletions. The frequency of these complex mutations was statistically higher in cases with longer (18 bp) losses: about 80% of double/multiple deletions resulted in a loss of 18 bps. Five novel mutations were observed by NGS in this study and all of them were complex, double/multiple mutations, that in 3 (60%) cases were not resolved by Sanger Sequencing. This clearly confirms the superiority of NGS in the characterization of *EGFR* microdeletions at exon 19. Rare cases of double/multiple deletions have been reported in previous studies, especially when conventional sequencing was conducted on multiple samples after cloning of genomic DNA into plasmids [19], [20], [31]. Cloning was essential to better characterize multiple deletions and rule out the possibility that they were present on different alleles. However, biological cloning and sequencing of multiple samples is time consuming and not suitable for routine clinical diagnostic purposes. The GS Junior Technology, based on PCR cloning before pyrosequencing of multiple samples, is in our opinion an ideal approach for a fine characterization of complex deletions in exon 19 of the *EGFR* gene.

The NGS assay is one of the most sensitive methods available for the detection of somatic mutations when used in deep sequencing, and the sensitivity of NGS is dependent on the number of sequences obtained per sample [25], [26]. In this study we decided to perform a deep NGS analysis taking a median of more than 3.000 sequences per sample. The high sensitivity of this NGS assay allowed us to detect in about 70% of cases, subpopulations of DNA molecules carrying exon 19 deletions different from the main mutation, but in most cases structurally related to it (Figure 4). In the majority of cases these subpopulations carried in frame simple or double deletions. In about one third of cases, less expanded non-in frame deletions were observed. These subpopulations were confirmed in independent PCR-NGS assays in 43% of the tumors investigated. However, only 4 tumors (4%) showed substantial subpopulations of deletions with at least one of them representing more than 2% of genomic DNA.

The presence of these subpopulations in a large proportion of cases examined is at moment unclear. It is extremely unlikely that they are due to cross-contaminations, since they were highly heterogeneous, usually related to the main deletion, and they were not seen in control samples. In addition, particular strategies were adopted to minimize cross-contaminations in our study (see Materials and Methods). These subpopulations could represent modifications of the main deletion or ex novo deletions acquired in cell clones during tumor progression. In both cases, this finding would support the hypothesis that this region within exon 19 of *EGFR* is highly instable in most patients affected by NSCLC carrying *EGFR* deletions. This genetic fragility may enable the development of both complex deletions and multiple subpopulations. To the best of our knowledge, this is the first demonstration of subpopulations of *EGFR* deletions in NSCLC. Our findings could have important clinical implications. Recent evidence indicate that tumor genotype may evolve dynamically under the selective pressure of targeted therapies [32]. We are tempted to hypothesize that the genetic fragility of exon 19 in particular patients could take a role in the dynamic evolution of lung tumors subjected to different therapeutic strategies. It would be interesting to monitor by NGS on repeated biopsies the main *EGFR* deletion as well as the deletions in minor clones during treatment.

In conclusion, our results indicate that NGS is particularly suitable for the study of *EGFR* deletions. This technique can accurately characterize *EGFR* deletions, even in cases in which conventional methods fail. Data obtained by NGS analysis allowed us to formulate a new interpretative hypothesis for complex deletions which represent about 20% of *EGFR* mutations in exon 19 as well as to identify 5 novel deletions. In addition, we report, for the first time to our knowledge, the presence of subpopulations of different deletions in most of the tumors investigated with pothential pathogenetic and clinical impact.

## Materials and Methods

### Patients and DNA samples

A series of 106 tumor DNA samples carrying *EGFR* deletions at exon 19, obtained from as many stage III–IV NSCLC patients, was collected in 3 reference diagnostic centers (Chieti, Rome, and Modena Centers). Genomic DNA was isolated from formaline-fixed, paraffin-embedded samples by standard procedures. Diagnostic evaluation of *EGFR* mutations was performed by High Resolution Melting Analysis (HRMA) followed by Sanger sequencing (Chieti Center), Fragment Analysis followed by Sanger sequencing (Rome Center) or direct Sanger Sequencing (Modena Center). Additional 10 NSCLC samples, found to be negative for deletions by Sanger Sequencing (control samples), were also available. All the DNA samples were subjected to deep sequencing by the 454 GS Junior System (454 Life Sciences, Branford, CT, and Roche Applied Sciences, Indianapolis, IN) in the Center of Predictive Molecular Medicine (University-Foundation, Chieti). Written consent was received by all patients. Researchers obtained permits from the diagnostic centers to use the tumor samples. All the samples were collected and received anonymously.

### PCR amplification

DNA fusions primers containing genome-specific sequences, along with one of 7 distinct 10-bp MIDs (multiplex identifier sequences used to differentiate samples being run together on the same plate) and sequencing adapters were used to amplify a 108 bp region in *EGFR* (NM_005228.3) exon 19 (Figure S1 and Table S1). PCR primers, were designed using the OligoAnalyzer 3.1 software (http://eu.idtdna.com/analyzer/Applications/OligoAnalyzer/) and synthesized at MWG-Biotech AG.

PCR reactions were run in 30 µl reaction volumes, containing 5.5 mmols dNTPs, 11 µmols of each primer, 2.75 µl PCR buffer, 1 µl DNA, and 1.3 units of FastStart HiFi Polymerase (Roche Diagnostics).

A touch-down PCR cycling program was performed on the Gene Amp PCR system 9700 thermocycler (Applied Biosystems) with an initial step at 94°C for 2 min followed by 43 cycles at 94°C for 30 sec, 64°C (decreasing the temperature by 1°C each cycle for six cycles) for 30 sec, and 70°C for 30 sec, and a final step at 70°C for 5 min. Different strategies were adopted to avoid cross-contaminations: a) reactions were set up in positive-pressure hoods with UV sterilization systems to decontaminate reagents and equipment prior to carrying out PCRs; b) different hoods were used for PCR amplification of samples subjected to different runs; c) PCR reactions were conducted on 96-well plates, with a maximum of 4 samples loaded per plate.

### Next Generation Sequencing

PCR products were visualized on agarose gel, purified using size-exclusion SPRI Ampure-XP DNA-binding paramagnetic beads (Agencourt Bioscience Corp., Beckman Coulter S.p.A, Milan, Italy), and quantified in 96-well format with the QuantiFluor™-ST Fluorometer (Promega, Madison, Wisconsin, USA) using a PicoGreen® assay (Invitrogen, Carlsbad, CA). Samples were then diluted to an approximate concentration of 1×10^9^ molecules/µL and pooled at equimolar concentrations to create a highly multiplexed amplicon library. After pooling, the library was further diluted to 10^6^ molecules/µl and subjected to emulsion PCR (emPCR) using the 454 GS Junior Titanium Series Lib-A emPCR Kit (Roche Diagnostics), according to the manufacturer's protocols. Following emPCR, the captured beads with bound DNA were enriched with a second DNA capture mechanism to separate out beads with and without bound emPCR products. By using a bead counter, the number of enriched beads was estimated to be between 300,000 and 1 million. The enriched pool of beads was then used for massively parallel pyrosequencing in a Titanium PicoTiterPlate® (PTP) with Titanium reagents (Roche Diagnostics), on the GS Junior instrument, according to the 454 GS Junior Titanium Series Amplicon Library Preparation Method Manual (available online: www.454.com).

### Analysis of sequence data

Processed and quality-filtered reads were analysed with the GS Amplicon Variant Analyzer (AVA) software version 2.5.3 (454 Life Sciences). *EGFR* exon 19 reference sequence was extracted from Hg19 Human Genome Version together with both neighbor intronic regions. Such sequence was used as Reference Sequence to align every reads. The final alignments were checked manually and wrong alignments were edited by Jalview Multi-alignment Tool (http://www.jalview.org/). In order to parse and manage all data sequences and results (variations, frequency of mutations, forward and reverse reads check) custom scripts were created using Perl language.

### Immunohistochemical analysis

The EGFR E746-A750 deletion specific (6B6) antibody (Cell Signaling Technology, Inc.) was used for immunohistochemical staining. Paraffin embedded sections were dewaxed, hydrated, treated with proteinase K (DAKO, Glostrup Denmark) and immunostained using a labelled polymer detection system (Bond Polymer Define Detection, Vision Biosystem, Mount Waverley, Australia) and automated stainer (BOND-maX, Vision BioSystem). The primary polyclonal antibody was used at a dilution of 1∶100. Negative controls were obtained by replacement of primary antiserum with buffer. IHC expression of mAbs against EGFR was evaluated using the following scoring: 0 = negative; 1 = weak staining in >10% of cancer cells; 2 = moderate staining in >10% of cancer cells; 3 = strong staining in >10% of cancer cells. A score of 0 was considered negative, a score of 1 was considered weakly positive, and a score of 2 or 3 was considered highly positive.

### Statistical analysis

The variables measured in the study were investigated for association by the Fisher's exact test or χ^2^ test as appropriate. A P<0.05 was considered as significant. Statistical analyses were performed using SPSS version 15 (SPSS, Chicago, IL).

## Supporting Information

Figure S1 **Polymerase chain reaction (PCR) primer design.** The squares represent the primer binding sequence, the circles represent the multiplex identifier (MID) sequence, and the thick lines represent the fusion primer sequence for 454 applications. The lenght of each primer is 61 and 57 nucleotides for forward and reverse, respectively. The total amplicon lenght is 178 bp including 108 bp of the *EGFR* gene.
(TIF)

Table S1 **Primer sequence for EGFR ex 19.** Starting at the 5′-end of each primer, fusion primer sequence for forward and reverse emulsion PCR and pyrosequencing is in standard font, followed by multiplex identifier sequence in italics and primer binding sequence in bold.
(DOC)

Table S2 **EGFR mutations observed by next generation sequencing in the whole series of tumors investigated.**
(XLS)
