# Supplementary figures and images for: Complex Mutations & Subpopulations of Deletions at Exon 19 of EGFR in NSCLC Revealed by Next Generation Sequencing: Potential Clinical Implications
Source: PLoS One. 2012 Jul 27;7(7):e42164. doi: 10.1371/journal.pone.0042164 (PMC3407088; doi:10.1371/journal.pone.0042164)

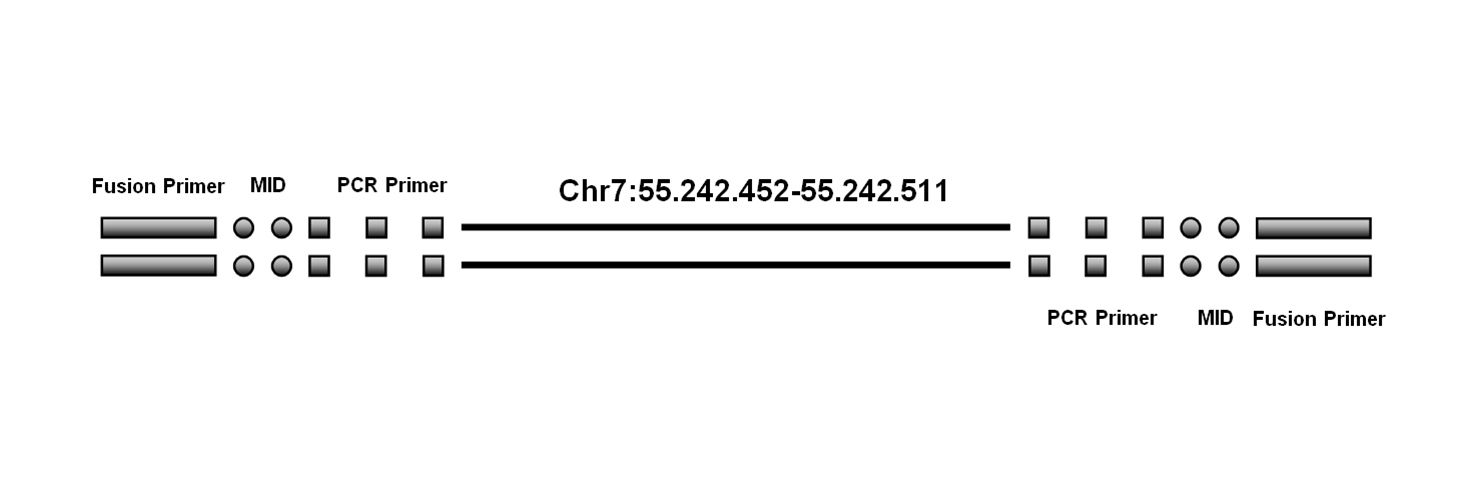

Supplement: Figure S1 — Polymerase chain reaction (PCR) primer design. The squares represent the primer binding sequence, the circles represent the multiplex identifier (MID) sequence, and the thick lines represent the fusion primer sequence for 454 applications. The lenght of each primer is 61 and 57 nucleotides for forward and reverse, respectively. The total amplicon lenght is 178 bp including 108 bp of the EGFR gene. (TIF) [file pone.0042164.s001.tif]
