# Supplementary material for: Complex Mutations & Subpopulations of Deletions at Exon 19 of EGFR in NSCLC Revealed by Next Generation Sequencing: Potential Clinical Implications
Source: PLoS One. 2012 Jul 27;7(7):e42164. doi: 10.1371/journal.pone.0042164 (PMC3407088; doi:10.1371/journal.pone.0042164)
Supplement: Table S1 — Primer sequence for EGFR ex 19. Starting at the 5′-end of each primer, fusion primer sequence for forward and reverse emulsion PCR and pyrosequencing is in standard font, followed by multiplex identifier sequence in italics and primer binding sequence in bold. (DOC) [file pone.0042164.s002.doc]

**Table S1**. Primer sequence for *EGFR* ex 19. Starting at the 5’-end of each primer, fusion primer sequence for forward and reverse emulsion PCR and pyrosequencing is in standard font, followed by multiplex identifier sequence in italics and primer binding sequence in bold.

| **Primer** | **Sequence** |
| --- | --- |
| MID1 forward | 5’-CGTATCGCCTCCCTCGCGCCATCAG *ACGAGTGCGT* **CCCAGAAGGTGAGAAAGATAAAATTC**-3’ |
| MID1 reverse | 5’-CTATGCGCCTTGCCAGCCCGCTCAG *ACGAGTGCGT* **ACAGCAAAGCAGAAACTCACAT**-3’ |
| MID2 forward | 5’-CGTATCGCCTCCCTCGCGCCATCAG *ACGCTCGACA* **CCCAGAAGGTGAGAAAGATAAAATTC**-3’ |
| MID2 reverse | 5’-CTATGCGCCTTGCCAGCCCGCTCAG *ACGCTCGACA* **ACAGCAAAGCAGAAACTCACAT**-3’ |
| MID3 forward | 5’-CGTATCGCCTCCCTCGCGCCATCAG *AGACGCACTC* **CCCAGAAGGTGAGAAAGATAAAATTC**-3’ |
| MID3 reverse | 5’-CTATGCGCCTTGCCAGCCCGCTCAG *AGACGCACTC* **ACAGCAAAGCAGAAACTCACAT**-3’ |
| MID4 forward | 5’-CGTATCGCCTCCCTCGCGCCATCAG *AGCACTGTAG* **CCCAGAAGGTGAGAAAGATAAAATTC**-3’ |
| MID4 reverse | 5’-CTATGCGCCTTGCCAGCCCGCTCAG *AGCACTGTAG* **ACAGCAAAGCAGAAACTCACAT**-3’ |
| MID5 forward | 5’-CGTATCGCCTCCCTCGCGCCATCAG *ATCAGACACG* **CCCAGAAGGTGAGAAAGATAAAATTC**-3’ |
| MID5 reverse | 5’-CTATGCGCCTTGCCAGCCCGCTCAG *ATCAGACACG* **ACAGCAAAGCAGAAACTCACAT**-3’ |
| MID6 forward | 5’-CGTATCGCCTCCCTCGCGCCATCAG *ATATCGCGAG* **CCCAGAAGGTGAGAAAGATAAAATTC**-3’ |
| MID6 reverse | 5’-CTATGCGCCTTGCCAGCCCGCTCAG *ATATCGCGAG* **ACAGCAAAGCAGAAACTCACAT**-3’ |
| MID7 forward | 5’-CGTATCGCCTCCCTCGCGCCATCAG *CGTGTCTCTA* **CCCAGAAGGTGAGAAAGATAAAATTC**-3’ |
| MID7 reverse | 5’-CTATGCGCCTTGCCAGCCCGCTCAG *CGTGTCTCTA* **ACAGCAAAGCAGAAACTCACAT**-3’ |
